# Exploring the Metabolic Pathways of Melon (*Cucumis melo* L.) Yellow Leaf Mutants via Metabolomics

**DOI:** 10.3390/plants14152300

**Published:** 2025-07-25

**Authors:** Fan Zhang, Kexin Chen, Dongyang Dai, Bing Liu, Yaokun Wu, Yunyan Sheng

**Affiliations:** 1 College of Horticulture and Landscape Architecture, Heilongjiang Bayi Agricultural University, Daqing 163000, China; zf1462434@126.com (F.Z.); 13846930436@163.com (K.C.); bobodaidy@163.com (D.D.); 2 Daqing Branch of Heilongjiang Academy of Agricultural Sciences, Daqing 163000, China; liubing528@163.com (B.L.); wuyaokun530@126.com (Y.W.)

**Keywords:** melon yellow leaf mutant ZT00091, carotenoid, chlorophyll, metabolomics, qRT-PCR

## Abstract

A yellow leaf mutant named ‘ZT00091’ was discovered during the cultivation of the melon variety ‘ZT091’. An analysis of the leaf ultrastructure revealed that the chloroplasts of ‘ZT00091’ were significantly smaller than those of ‘ZT091’, with irregular shapes, blurred contours, and no starch granules. Metabolomic analysis revealed 792 differentially abundant metabolites between ‘ZT00091’ and ‘ZT091’, with 273 upregulated and 519 downregulated. The Kyoto Encyclopedia of Genes and Genomes (KEGG) results indicated that the differentially abundant metabolites were enriched mainly in the carotenoid pathway. qRT-PCR was used to analyze key genes in the carotenoid pathway of melon. Compared with those in ‘ZT091’, the genes promoting carotenoids and lutein in ‘ZT00091’ were significantly upregulated, which may explain the yellow color of ‘ZT00091’ leaves. Significant differences in the chlorophyll contents (chlorophyll a, chlorophyll b, and total chlorophyll) and carotenoid contents were found between ‘ZT00091’ and ‘ZT091’, indicating that the yellowing of melon leaves is related to changes in the carotenoid and chlorophyll contents. This study provides a theoretical basis for research on the molecular mechanism of melon yellowing.

## 1. Introduction

Melon (*Cucumis melo* L.) is very popular among consumers because of its sweet, delicious, and delicate taste and high nutritional value, among other characteristics [1]. Photosynthesis can convert carbon dioxide and water into organic compounds such as glucose, promoting the accumulation of sugar in melon fruits, and the color of the leaves largely determines the photosynthetic capacity of the melon [2]. At present, various colored leaf mutants, such as emerald green, yellow-green, striped, yellow, and white, have been found in plants [3]. Although the chlorophyll content of yellow leaves is relatively low and the photosynthetic rate decreases, leading to dwarfing, yield reduction, mortality, and severe damage to crop yield and product quality [4], yellow leaf mutant plants are good genetic breeding materials and can play an important role in the selection of specific germplasm resources [5]. In recent years, the literature on Cucurbitaceae leaf color mutants has focused mainly on watermelon and pumpkin, and the molecular mechanism underlying the appearance of yellow leaf mutants in melon remains unclear.

Researchers have discovered different types of leaf color mutants from rice [6], wheat [7], soybean [8], corn [9], cucumber [10], and some vegetable crops. Through in-depth studies on these leaf color mutants, the value of yellow leaf mutants has gradually been discovered. Metabolomics can be used to analyze small organic molecules, also known as metabolites, both qualitatively and quantitatively in plants, allowing for accurate and efficient determination of differences in these molecules among different biological varieties [11,12]. Yellowing is often accompanied by disturbances in the metabolic pathways of carotenoids, chlorophyll, and reactive oxygen species (ROS). Metabolomics can locate specific blocked links, clarify the molecular mechanism of yellowing, and accurately detect specific metabolites that accumulate or are reduced during leaf yellowing, such as carotenoids, chlorophyll, coumarin, and oxalic acid, which may directly participate in stress resistance or yellowing control in melons [13,14]. Wang et al. performed a metabolomic analysis and reported that the metabolism of various redundant pigments, such as chlorophyll, carotenoids, and flavonoids, in plants has a significant effect on leaf color [15]. Wu et al. performed metabolomic analysis and revealed that there are 50 metabolites whose levels differ between the Ginkgo biloba yellow leaf mutant and the wild type; these metabolites are enriched mainly in the chlorophyll, flavonoid, and carotenoid pathways [16]. These findings indicate that the molecular mechanism of yellow leaf formation in plants is regulated by its own complex metabolic network.

Carotenoids are key pigments in the orange-yellow to orange-red color of melon flesh, and their types and contents directly affect the color differences in different varieties. β-carotene and lutein are key metabolites in the metabolic pathway of sweet melon. β-Carotene is a precursor of vitamin A with antioxidant activity, which can clear free radicals and enhance human immunity. Lutein has potential benefits for vision protection and skin health and can increase the market competitiveness of cantaloupe [17,18,19]. The precursor substance of carotenoids in muskmelon, *GGPP*, is formed by the MEP pathway through the catalysis of isopentene pyrophosphate (IPP) and 3,3-dimethylpropenyl pyrophosphate (DMAPP) by *GGPP* synthase [20]. Two molecules of *GGPP* generate eight hydrolycopenes (phytoenes) under the action of eight hydrolycopene synthases (*PSYs*) [21]. Under the action of carotene deoxygenation and enzymes (*ZDS*), lycopene is ultimately generated. Lycopene is used as a precursor substance to enter two parallel synthesis pathways, and the first pathway involves the lycopene β-catalyzed generation of the cyclizing enzyme β-carotene. *CRTR-B* catalyzes its formation as zeaxanthin, which then combines with *VDE* and *ZEP* to form violaxanthin. Violaxanthin is reduced by *NXS* to form neoxanthin or is oxidized to form xanthins, ultimately synthesizing ABA. In the second pathway, lycopene ε is used under the joint action of cyclinase (*LCYE*) and *LCYB* α-carotenoids [22,23]. α-Carotene forms from lutein under the catalysis of *CRTR-E*. Carotenoids, violaxanthin, neoxanthin, and lutein have been confirmed to change the accumulation of pigments in plants and regulate leaf color changes [24]. Previous studies have investigated the physiological characteristics, genetic characteristics, and proteomic changes in the leaves of melon yellow leaf mutants. However, owing to the lack of reported metabolomic information on the yellowing of melon leaves, little is known about the mechanism of yellow melon leaf formation.

In our group’s prior research, the genetic pattern of yellowing was studied through gene mapping, and it was found that the key functional gene was located in the range of 23,143,515 to 24,076,164 on chromosome 11. A hybrid combination was configured and used to observe the population of six generations. It was found that the yellowing trait is regulated by a pair of recessive genes. Years of field phenotype observation have found that its yellow leaf phenotype is heritable. In this study, melon yellow leaf mutant ‘ZT00091’ and wild-type ‘ZT091’ plants were experimental materials that had been self-pollinated for 4 years. The differences in cell structure between ‘ZT00091’ and ‘ZT091’ were observed using transmission electron microscopy, the nontargeted metabolites were analyzed using metabolomics, and the differentially abundant metabolites were identified and analyzed using KEGG enrichment. qRT-PCR was used for the analysis of key genes in the carotenoid pathway of melon. Chlorophyll a, chlorophyll b, carotenoids, flavonoids, total phenols, and other leaf pigment-related indices of melon ‘ZT00091’ yellow leaf mutant and wild-type plants were detected. This study aims to provide a reference for further research on the metabolic mechanism of yellow melon leaves and new materials for the production and application of melon.

## 2. Materials and Methods

### 2.1. Materials

**Figure 1:**
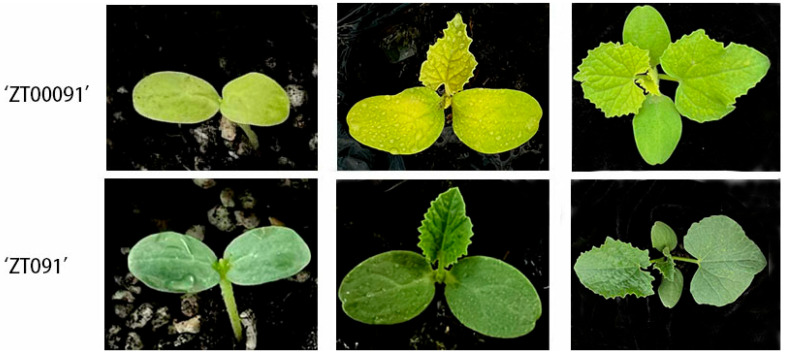
Phenotypes of the yellow-leaf mutant ‘ZT00091’ and the melon variety ‘ZT091’.

‘ZT00091’ (yellow leaf mutant) and ‘ZT091’ (parental melon variety) were used as the experimental materials. The seeds were provided by the College of Horticulture and Landscape Architecture of Heilongjiang Bayi Agricultural University, Daqing, China (45°46′ N, 124°19′ E). ‘ZT00091’ and ‘ZT091’ were planted in Biotron in March 2021, with temperatures of 30 °C day/20 °C night day, 16 h day/8 h night, and 65% relative humidity in the field. Each planting area was 14.4 m^2^, including four 0.6 × 0.6 m rows of melons planted. The experiment was performed in accordance with a randomized block design and was replicated three times. In the field, the native soil contained 5.39 g/kg organic substrate, the pH was 7.9, and the available nitrogen, phosphorus, and potassium (mg/kg) in the field were 44.76, 17.51, and 94.75, respectively. The leaf ultrastructures of three-leaf-stage ‘ZT00091’ and ‘ZT091’ plants were observed. Once the melons had reached the five-leaf stage, the fresh leaves of ‘ZT00091’ and ‘ZT091’ were cut and quickly frozen in liquid nitrogen for metabolomic analysis, after which the pigment content was determined. The experiment was repeated three times (Figure 1).

### 2.2. Methods

#### 2.2.1. Observation of the Ultrastructural Morphology of Chloroplasts

The fully developed functional leaves of ‘ZT00091’ and ‘ZT091’ at the three-leaf stage were selected for analysis using transmission electron microscopy. Small squares with a size of 1~2 mm were cut and immediately fixed in 2.5% glutaraldehyde. After the samples were fixed overnight at 4 °C, they were washed three times with phosphoric acid buffer solution at a pH of 7.2 for 30 min, fixed for 2 h in 1% osmic acid, dehydrated step by step with ethanol, infiltrated with an acetone mixture, and embedded in epoxy resin 812. An ultrathin ULPRACUT-E slicing machine (Leica Microsystems, Wetzlar, Germany) was used for sectioning and staining (lead citrate and uranyl acetate) [25], and the ultrastructure was observed via transmission electron microscopy (H-7650).

#### 2.2.2. Sample Extraction

The experiment was based on an LC-MS technique to detect metabolites in melon leaf samples with different leaf colors. The ‘ZT00091’ and ‘ZT091’ samples were taken at the five-leaf stage and divided into 2 experimental groups, each with 3 replicates. The yellow-leaf mutant ‘ZT00091’ is represented by Y1, Y2, and Y3, whereas the ‘ZT091’ wild-type plants are represented by H1, H2, and H3. The metabolomics material was also sent to Beijing BioMarker Company (Beijing, China) for sequencing.

Leaf samples (50 mg) were accurately weighed, and 1000 μL of extraction solution containing an internal standard (methanol: acetonitrile: water volume ratio = 2:2:1, internal standard concentration of 20 mg/L) was added. The mixture was vortexed for 30 s. Steel balls were added to the sample, which was then ground with a 45 Hz grinder for 10 min and sonicated for 10 min (in an ice/water bath). The samples were subsequently prepared for metabolomic analysis.

The LC-MS system for metabolomic analysis consists of a Waters Acquity I-Class PLUS (Waters, Milford, MA, USA) ultrahigh-performance liquid tandem Waters Xevo G2-XS QToF high-resolution mass spectrometer. The UPLC HSS T3 column (1.8 µm 2.1 × 100 mm) used was purchased from Waters Acquity (Milford, MA, USA). Both the positive and negative ion mode conditions were as follows: mobile phase A was a 0.1% formic acid aqueous solution, and mobile phase B was a 0.1% formic acid in acetonitrile. The injection volume was 1 μL.

#### 2.2.3. Metabolomic Analysis

The Waters Xevo G2-XS QToF high-resolution mass spectrometer was be operated under the control of acquisition software (MassLynx V4.2, Waters, Milford, MA, USA). The MSe mode was used for MS1 and MS2 mass spectrometry data collection. In each data acquisition cycle, dual-channel data acquisition can be performed simultaneously for low and high collision energies. The low collision energy was 2 V, and the high collision energy range was 10–40 V. The quality control materials were mixed equally with two leaf extracts and tested using the same method as the analysis samples. The mass spectrum was scanned at a frequency of 0.2 s. The ESI ion source parameters were as follows: (1) capillary voltage, 2500 V (positive ion mode) or −2000 V (negative ion mode); (2) cone hole voltage, 30 V; (3) ion source temperature, 100 °C; (4) desolvent gas temperature, 500 °C; (5) blowback flow rate, 50 L/h; (6) desolvent gas flow rate, 800 L/h; and (7) mass-to-charge ratio (*m*/*z*) collection range of 50–1200.

The raw data collected using MassLynx V4.2 were processed using Progenesis QI V3.0 software for peak extraction, peak alignment, and other data processing operations. On the basis of the Progenesis QI software online METLIN database, public database, and custom-built database of Baimaike, identification was carried out, and theoretical fragment identification was also performed. The deviation of the parent ion mass number was within 100 ppm, and the deviation of the fragment ion mass number was within 50 ppm.

After the original peak area information was normalized to the total peak area, follow-up analysis was performed. Principal component analysis (PCA) and Spearman correlation analysis were used to judge the repeatability of the samples within groups and the quality of the control samples. The identified compounds were searched for classification and pathway information in the KEGG, HMDB, and lipidmaps databases. According to the grouping information, the difference multiples were calculated and compared, and a t test was used to calculate the difference significance *p* value of each compound. The R language package 4.1.3 ropls was used to perform orthogonal partial least squares discriminant analysis (OPLS-DA) modeling, and 200 times permutation tests were performed to verify the reliability of the model. The VIP value of the model was calculated using multiple cross-validations. The method of combining the difference multiple, *p* value and VIP value of the OPLS-DA model was adopted to screen the differentially abundant metabolites. The screening criteria were FC ≥ 2, *p* value < 0.05 and VIP ≥ 1. The differentially abundant metabolites associated with KEGG pathway enrichment significance were calculated via a hypergeometric distribution test.

#### 2.2.4. Expression Pattern Analysis

RNA was extracted from 0.1 g of melon yellow leaf mutant and wild-type leaves via the TRIzol method and reverse transcribed into cDNA. The cDNAs of ‘ZT00091’ and ‘ZT091’ were used as templates. The online tool Primer3plus was used for primer design, and the primers used are shown in Table 1. *EF1α* (GenBank accession number: XM_008459007.2) was used as the reference gene [26]. The experiment included a total of three biological replicates, and the relative expression level was determined via the 2^−ΔΔCT^ relative quantitative analysis method [27]. Student’s *t* test was used for sample comparison.

#### 2.2.5. Determination of the Pigment Content

After the veins were removed from ‘ZT00091’ and ‘ZT091’, three 1.0 g samples of melon leaves were weighed. Quartz sand, calcium carbonate powder, and 20 mL of 95% ethanol were added during grinding. The sample was then transferred to a 50 mL centrifuge tube and left in the dark for 10 min. The sample was subsequently centrifuged at room temperature at a speed of 4000 r/min for 10 min, after which the supernatant was extracted and brought to 25 mL [28].

An ultraviolet spectrophotometer (UV-2550) was used to detect the absorbance at 663, 645, and 470 nm. Each sample was measured three times, and the average value was used in the content determination formula to calculate the content. The following formulas were used:

chlorophyll a concentration (mg·L): Ca = 12.72A663 − 2.59A645;

chlorophyll b concentration (mg·L): Cb = 22.88A645 − 4.67A663;

total chlorophyll concentration (mg·L): CT = Ca + Cb = 20.29A645 − 8.05A663;

carotenoid concentration (mg·L): C = (1000A470 − 3.27Ca − 104Cb)/229.

pigment content (mg·g) = [pigment concentration (mg/L) × extract volume (L) × dilution ratio]/sample weight (g).

After the veins were removed from the fresh leaves of ‘ZT00091’ and ‘ZT091’, three 0.5 g samples were weighed and placed into a mortar with a small amount of precooled 1% HCl methanol solution, ground into a pulp in an ice bath, shaken, and extracted at 4 °C in the dark for 20 min. The sample was shaken several times during this period. The sample was subsequently filtered, and the filtrate was collected for use.

The sample solution and 1% HCl methanol solution were used as blanks. The total phenolic and flavonoid contents were measured using spectrophotometry. An ultraviolet spectrophotometer (UV-2550) was used to detect the absorbance at 280 nm and 325 nm. The absorbance was measured three times for each sample, and the average value was used in the content determination formula to calculate the content. The formulas are as follows: total phenolics (μmol/g): A280/g; flavonoid content (μmol/g): A325/g [29].

### 2.3. Statistical Analysis

OPLS-DA was used to analyze ‘ZT00091’ and ‘ZT091’, and Vip > 1 and *p* < 0.05 were used as thresholds to determine the significantly different metabolites. Using the “ggplot2” software package of R, the differentially abundant metabolites between the two groups of ‘ZT00091’ etiolated mutant and normal plants were analyzed. The “clusterprofiler” software package of R was used for KEGG enrichment analysis of the differentially abundant metabolites. Origin 8.0 and DPS 9.5 software were used for data analysis, and all data measurements were replicated at least three times. The “∗” represents *p* < 0.05; the “∗∗” represents *p* < 0.01.

## 3. Results

### 3.1. Cell Structure of Chloroplasts in Leaves with Different Colors

Field phenotypic observations revealed that the leaf color of ‘ZT00091’ at the seedling stage was etiolated, the plant was thin and weak, the growth was slow, the growth period was prolonged, and fewer male flowers and more female flowers existed. The color of the leaves of ‘ZT091’ was green, the plants were large and thick in texture, and the stems were robust with normal growth. With increasing plant growth, the difference in etiolation became increasingly obvious. The yellow leaf mutant is a nonlethal mutant that does not easily bear fruit and has poor economic traits.

**Figure 2:**
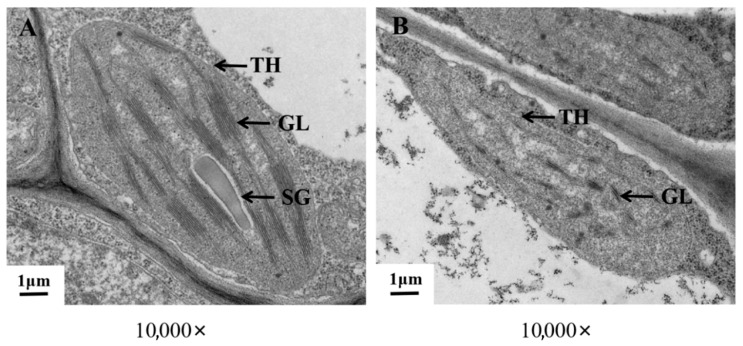
Ultrastructure of chloroplasts in ‘ZT00091’ and ‘ZT091’. (**A**) Wild-type plant leaves; (**B**) yellow leaf mutant plant leaves. TH, Chloroplast; GL, substrate layer; SG, starch granule.

To explore whether chloroplasts play a role in melon leaf color mutation, we observed the cell structure of the mutant (Figure 2) and found that the chloroplasts in the mesophyll cells of ‘ZT00091’ were infrequent and small, with irregular long spindle shapes and unclear outlines. The grana lamellae were loosely arranged and scattered, with large gaps between lamellae, unclear layers, or even broken and shapeless, and no starch grains were observed in the chloroplasts. The chloroplast structure of ‘ZT091’ was normal, and the growth status was good. Most chloroplasts were long oval or spindle-shaped, the chloroplast envelope was complete, the lamellar structure was stacked neatly, the grana lamellae were arranged compactly and clearly, and the starch grains in the chloroplasts were abundant and large. The integrity of chloroplasts is believed to be one of the key factors leading to color alterations in melon leaves.

### 3.2. Pigment Contents

**Figure 3:**
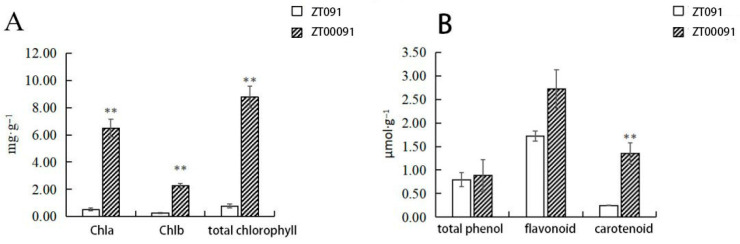
Comparison of the pigment contents of yellow leaves of the ‘ZT00091’ and ‘ZT091’ cultivar plants (** *p* < 0.01); the error line represents the standard deviation (SD). Chla, Chlorophyll (**A**); Chlb, Chlorophyll (**B**).

The chlorophyll a, chlorophyll b, carotenoid, total phenol, and flavonoid contents of ‘ZT00091’ were 0.51 mg·g^−1^, 0.26 mg·g^−1^, 0.24 mg·g^−1^, 0.79 μmol·g^−1^ and 1.72 μmol·g^−1^, respectively. The chlorophyll a, chlorophyll b, carotenoid, total phenol, and flavonoid contents of ‘ZT091’ were 6.50 mg·g^−1^, 2.27 mg·g^−1^, 1.35 mg·g^−1^, 0.88 μmol·g^−1^, and 2.72 μmol·g^−1^, respectively (Figure 3). The results revealed different leaf color chlorophyll contents (chlorophyll a, chlorophyll b, and total chlorophyll contents) and carotenoid contents. Through the above comparative analysis, this study preliminarily suggested that the formation of the leaf phenotype in etiolated plants may be related to the significant decrease in the total chlorophyll content and Chla/b value and the significant increase in the proportion of carotenoids.

### 3.3. Yellow Leaf Mutant Metabolomic Analysis

#### 3.3.1. Analysis of Nontargeted Metabolites via PCA and OPLS-DA

**Figure 4:**
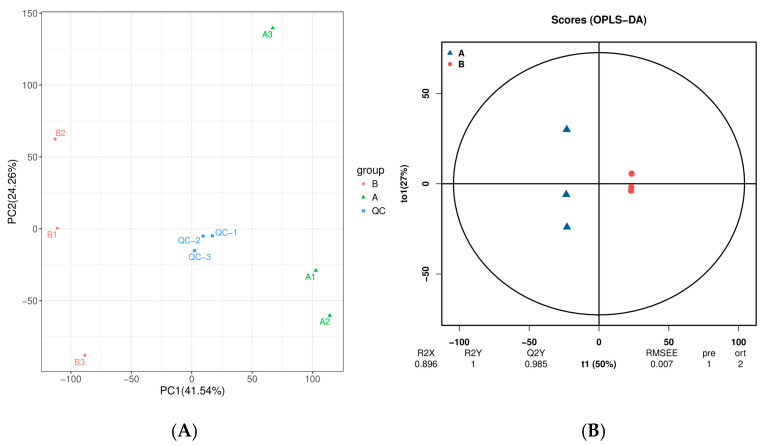
Nontargeted metabolite analysis Via PCA and OPLS-DA: (**A**) PCA scatter plot and (**B**) OPLS-DA score graph.

To explore the differences in metabolite composition between ‘ZT00091’ and ‘ZT091’, we measured and analyzed their metabolomes. The PCA results of ‘ZT00091’ are presented on the right side (A1, A2, and A3), and those of ‘ZT091’ are presented on the left side (B1, B2, and B3), with clear distinctions between them. The metabolic phenotypes of ‘ZT00091’ and ‘ZT091’ significantly differed. The results revealed significant differences in metabolites between ‘ZT00091’ and ‘ZT091’, and the difference in metabolites within the group was less than that between the groups. The contribution rate of PC1 was 41.54%, and that of PC2 was 24.26%. The sample data were highly reliable and applicable for subsequent differentially abundant metabolite analysis (Figure 4A).

To display the metabolic differences more accurately between the two melon plants, the least squares method (PLS-DA) model was used for further analysis. The core value (T1) and orthogonal value (to1) were 50% and 27%, respectively. Here, r2x = 0.896, r2y = 1, and q2y = 0.985; R2y and q2y are greater than 0.5, indicating that the reliability of the model is good and that the model can effectively explain and predict the difference between ‘ZT00091’ and ‘ZT091’. The OPLS-DA results are shown in Figure 4B. The normal plant samples and yellow leaf plant samples were clearly distinguished.

### 3.3.2. Screening and Identification of Differentially Abundant Metabolites

**Figure 5:**
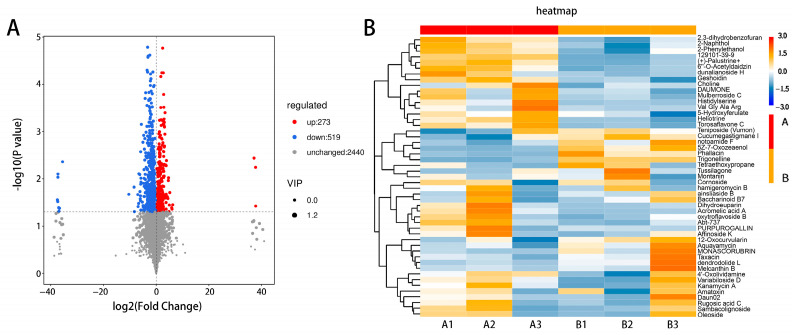
Screening and identification of differentially abundant metabolites: (**A**) volcano map of differentially abundant metabolites and (**B**) heatmap of metabolites.

After testing, a total of 3232 metabolites produced in the leaves of ‘ZT00091’ and ‘ZT091’ (Table S1) were identified in ‘ZT00091’ and ‘ZT091’, of which 792 were differentially abundant. Compared with those in ‘ZT091’, 273 metabolites were upregulated and 519 were downregulated in ‘ZT00091’ (Figure 5A). The greatest difference between ‘ZT00091’ and ‘ZT091’ is the difference in leaf color, which is reflected in the significant differences in the metabolites between them. The cluster heatmap of the metabolites is in Figure 5B, including 50 types such as 2,3-dihydrobenzofuran, naphthol, geshoidin, and choline, etc. The levels of nonmetallic oxygen-negative compounds and pregnenolone lipids in ‘ZT00091’ were significantly greater than those in ‘ZT091’, whereas the organic oxygen and fatty acyl contents were significantly lower than those in ‘ZT091’. These findings indicate that these metabolites may be associated with the regulatory mechanism of melon leaf yellowing.

### 3.3.3. KEGG Enrichment Analysis of Differentially Abundant Metabolites

**Figure 6:**
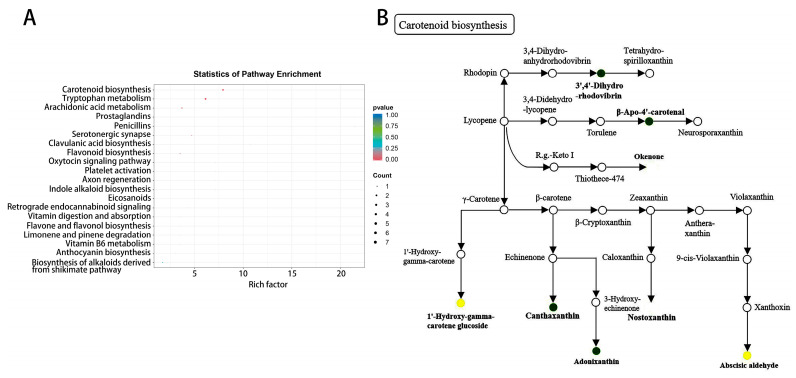
KEGG enrichment analysis of differentially abundant metabolites. (**A**) Bubble plot of differentially abundant metabolites. (**B**) Annotation diagram of the carotenoid biosynthesis pathway. Note: Dark green represents an increase in metabolite content, bright Yellow represents a decrease in metabolite content.

The KEGG enrichment analysis results indicated that the key differentially abundant metabolites were enriched in 20 metabolic pathways, which were mainly enriched in pathways such as carotenoid biosynthesis, tryptophan metabolism, arachidonic acid metabolism, and flavonoid biosynthesis (Figure 6A). After further analysis, six metabolites with significant differences were found in the carotenoid biosynthesis pathway (Figure 6B), such as canthaxanthin. Therefore, the change in melon leaf color could be due mainly to an increase in carotenoids.

### 3.4. Expression Levels of Genes in the Carotenoid Pathway

**Figure 7:**
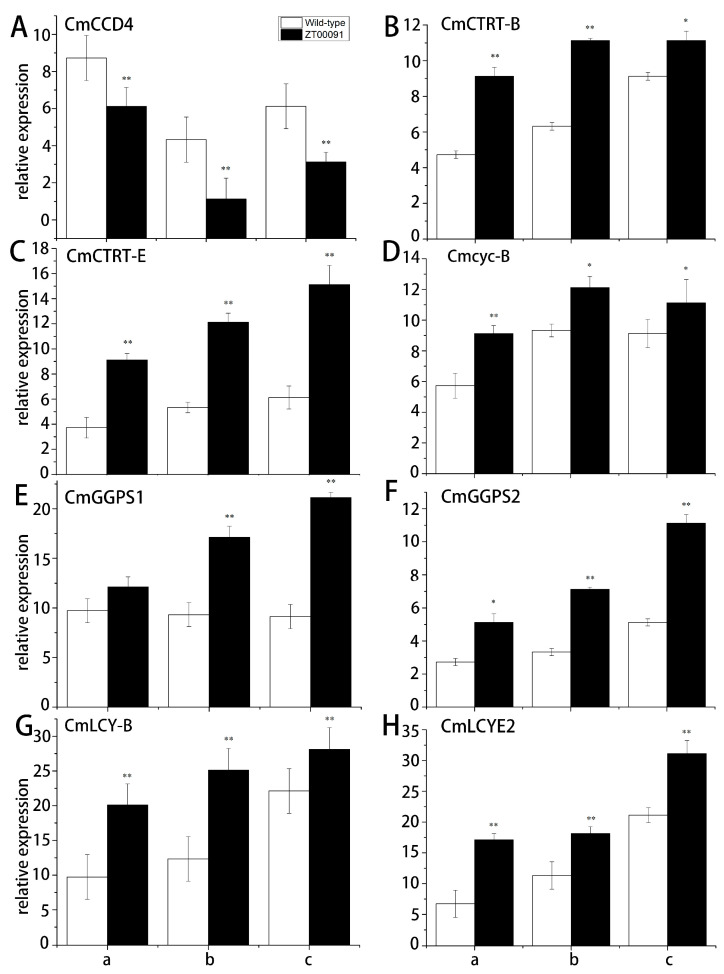
Relative expression patterns of key genes in the carotenoid pathway. Note: a: 2-leaf stage; b: 4-leaf stage; and c: 6-leaf stage; (**A**): The expression level of *CmCCD4* in ‘ZT00091’ and wild-type of ZT091; (**B**):The expression level of *CmCTRT-B* in ‘ZT00091’ and wild-type of ZT091; (**C**):The expression level of *CmCTRT-E* in ‘ZT00091’ and wild-type of ZT091; (**D**):The expression level of *Cmcyc-B* in ‘ZT00091’ and wild-type of ZT091; (**E**):The expression level of *CmGGPS1* in ‘ZT00091’ and wild-type of ZT091; (**F**):The expression level of *CmGGPS2* in ‘ZT00091’ and wild-type of ZT091; (**G**):The expression level of *CmLCY-B* in ‘ZT00091’ and wild-type of ZT091; (**H**):The expression level of *CmLCYE2* in ‘ZT00091’ and wild-type of ZT091; “*” indicates that *p* < 0.05; “**” indicates that *p* < 0.01; the error line represents the standard deviation (SD).

The relative expression patterns of the genes in the carotenoid biosynthesis pathway were analyzed through qRT-PCR (Figure 7). The relative expression of *CmCCD4* first increased but then decreased, and its expression level in ‘ZT00091’ was 0.67-, 0.76-, and 0.53-fold lower than that in ‘ZT091’ (Figure 7A). The expression level of *CmCRTR-B* in ‘ZT00091’ at all growth stages was significantly greater (1.65-, 1.75-, and 1.21-fold greater, respectively) than that in the other variety (Figure 7B). Compared with that in ‘ZT091’, the relative expression of *CmCRTR-E* in ‘ZT00091’ at all growth stages was significantly upregulated, with values that were 2.46-, 2.27-, and 2.50-fold greater, respectively (Figure 7C). At the 2-leaf stage, the expression level of *Cmcyc-B* in ‘ZT00091’ was 1.58-fold greater than that at the 4-leaf and 6-leaf stages, and the *Cmcyc-B* expression level in ‘ZT00091’ was 1.30- and 1.22-fold greater than that in ‘ZT091’, respectively (Figure 7D). The relative expression of *CmGGPS1* in ‘ZT00091’ at the 4-leaf and 6-leaf stages was much greater (by 1.59- and 2.34-fold, respectively) than that in ‘ZT091’. No significant difference in the expression level of *CmGGPS1* was found between ‘ZT00091’ and ‘ZT091’ at the 2-leaf stage (Figure 7E). At the 2-leaf stage, the expression level of *CmGGPS2* in ‘ZT00091’ was significantly greater (by 1.83-fold) than that in ‘ZT091’ and was 2.18- and 2.08-fold greater than that in ‘ZT00091’ at the 4-leaf and 6-leaf stages, respectively (Figure 7F). *CmLCY-B* expression steadily increased with growth and long-term changes, and the expression level in ‘ZT00091’ during each stage was significantly greater than that in ‘ZT091’, with values 2.03-, 2.02-, and 1.24-fold greater than those in the other stages, respectively (Figure 7G). At three different growth stages (the 2-leaf, 4-leaf, and 6-leaf stages), the *CmLCYE2* expression level in ‘ZT00091’ was 2.48-, 1.61-, and 1.45-fold greater, respectively, than that in ‘ZT091’ (Figure 7H).

**Figure 8:**
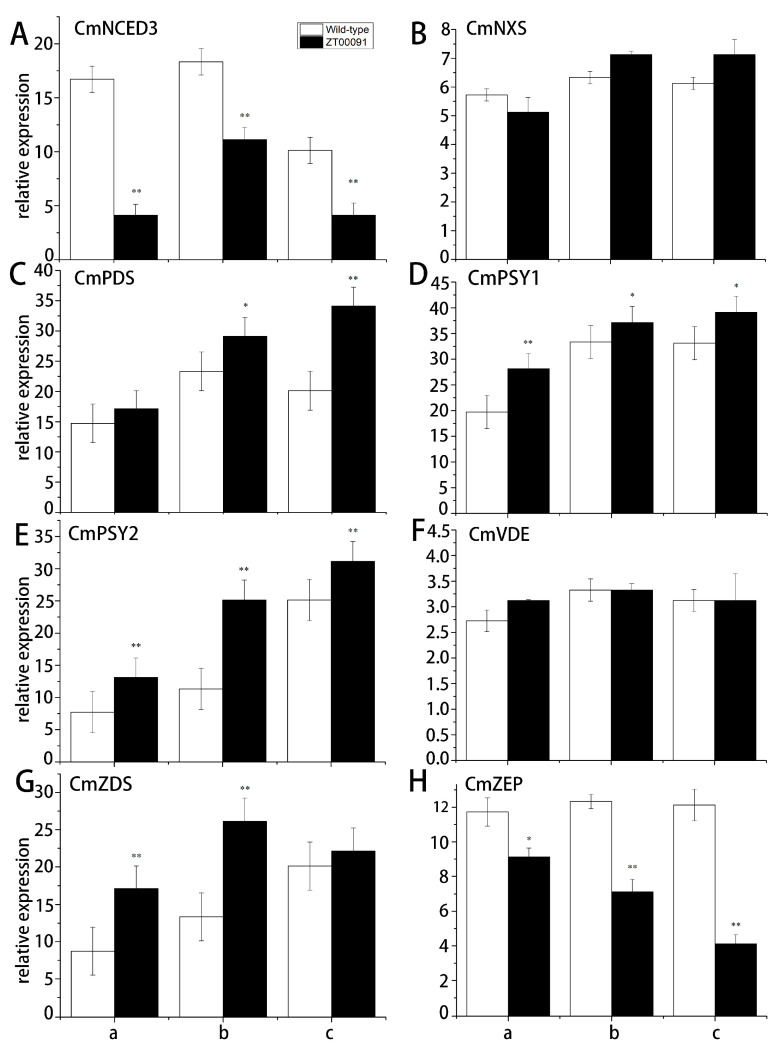
Relative expression patterns of key genes in the carotenoid pathway. Note: a: 2-leaf stage; b: 4-leaf stage; and c: 6-leaf stage; (**A**): The expression level of *CmNCED3* in ‘ZT00091’ and wild-type of ZT091; (**B**): The expression level of *CmNXS* in ‘ZT00091’ and wild-type of ZT091; (**C**): The expression level of *CmPDS* in ‘ZT00091’ and wild-type of ZT091; (**D**): The expression level of *CmPSY1* in ‘ZT00091’ and wild-type of ZT091; (**E**): The expression level of *CmPSY2* in ‘ZT00091’ and wild-type of ZT091; (**F**): The expression level of *CmVDE* in ‘ZT00091’ and wild-type of ZT091; (**G**): The expression level of *CmZDS* in ‘ZT00091’ and wild-type of ZT091; (**H**): The expression level of *CmZEP* in ‘ZT00091’ and wild-type of ZT091; “*” indicates that *p* < 0.05; “**” indicates that *p* < 0.01; the error line represents the standard deviation (SD).

The expression level of *CmNCED3* at all stages in ‘ZT00091’ was significantly lower than that in the wild type; the expression level peaked at the 4-leaf stage, which was 0.70-fold greater than that of ‘ZT091’ and presented a normal distribution (Figure 8A). No significant difference in the relative expression of *CmNXS* was found in ‘ZT00091’ and ‘ZT091’ during different growth stages, with expression levels ranging from 5.61 to 7.05 (Figure 8B). The relative expression of *CmPDS* at the 2-leaf stage did not significantly differ between ‘ZT091’ and ‘ZT00091’. When the plants were grown to the 4-leaf stage, *CmPDS* was significantly upregulated in ‘ZT00091’ and was 1.25-fold greater than that in the wild type. At the 6-leaf stage, the expression of *CmPDS* was highly upregulated in ‘ZT00091’ and was 1.79-fold greater than that in ‘ZT091’ (Figure 8C). At the 2-leaf stage, the relative expression of *CmPSY1* was significantly greater (by 1.40-fold) than that of ZT091. The difference between the 4-leaf stage and the 6-leaf stage decreased but was still significantly greater (by 1.11-fold and 1.13-fold, respectively) than that of the ‘ZT091’ cultivar (Figure 8D). The relative expression of *CmPSY2* in ‘ZT00091’ was highly upregulated and was 1.82-fold, 2.19-fold, and 1.20-fold greater than that in ‘ZT091’ (Figure 8E). No significant difference in the relative expression of *CmVDE* was found between ‘ZT00091’ and ‘ZT091’ during different growth stages, with expression levels ranging from 2.72 to 3.34, indicating a linear distribution (Figure 8F). During the 2-leaf and 4-leaf stages, the relative expression of *CmZDS* in ‘ZT00091’ significantly increased and was 1.95- and 2.03-fold greater than that in ‘ZT091’, respectively. However, no difference in its expression level was found between ‘ZT00091’ and ‘ZT091’ at the 6-leaf stage (Figure 8G). At the 2-leaf stage, the expression level of *CmZEP* was significantly lower in ‘ZT00091’ than in ‘ZT091’, which was 1.31-fold greater than that in the other varieties and was significantly greater at the 4-leaf and 6-leaf stages (1.74- and 2.98-fold greater than that in ‘ZT091’, respectively) (Figure 8H).

### 3.5. ‘ZT00091’ Pattern Diagram of the Carotenoid Synthesis Pathway

**Figure 9:**
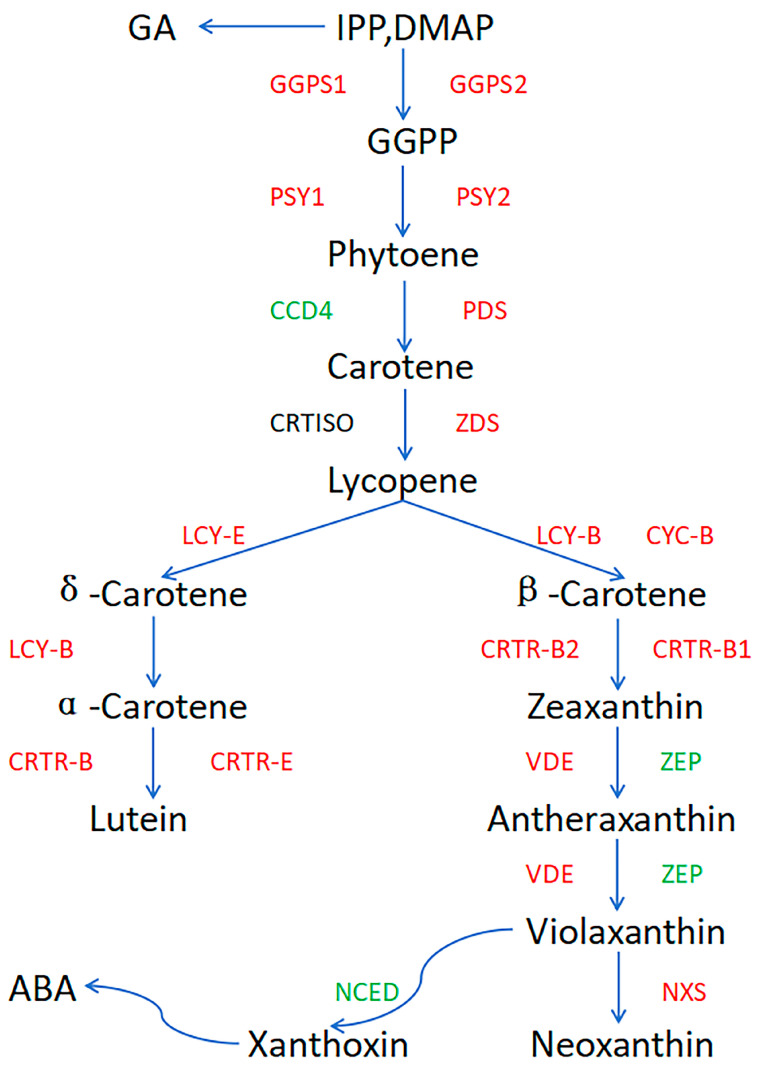
Pattern diagram of the carotenoid synthesis pathway in ‘ZT00091’. Note: red represents upregulation of the gene, green represents downregulation of the gene.

Expression pattern analysis was conducted on 16 genes involved in the carotenoid biosynthesis pathway (Figure 9), including the genes involved in the MEP pathway that converts IPP and DMAP to lycopene (*CmGGPS1*, *CmGGPS2*, *CmPSY1*, *CmPSY2*, *CmPDS*, *CmCCD4*, and *CmZDS*), some genes involved in the lycopene to xanthoxin pathway (*CmLCY-B*, *CmCYC-B*, *CmCRTR-B*, *CmVDE*, *CmZEP*, *CmNXS*, and *CmNCED3*), and some genes involved in the lycopene to lutein pathway (*CmLCY-B*, *CmCRTR-B*, *CmLCYE2*, and *CmCRTR-E*).

## 4. Discussion

Yellow leaf mutants have been found in many plants. Among these mutants, the yellowing characteristic of most plants is not stable and changes with changes in the growth period [30,31,32]. Xue Fei discovered a cotton leaf yellowing mutant, SD18-46, in a natural population, which was caused by the deletion of *GhSCY2D* on the 12th chromosome, resulting in lower chlorophyll content and underdeveloped chloroplast structure in the mutant compared to the wild type. The expression levels of key genes involved in chlorophyll synthesis were significantly downregulated [33]. The 186th amino acid mutation in the *CHLI* gene of the strawberry leaf color mutant (p240) leads to damage to chloroplasts and inhibition of chlorophyll synthesis [34]. Natural mutants of plants suffer from the low chlorophyll content, the photosynthetic rate decreases, resulting in dwarfing, yield reduction and death. The content of soluble sugars, RUBP carboxylase (RUBPCase), sucrose synthase (SS), and sucrose phosphate synthase (SPS) in the leaves of rice yellow leaf mutant (Huangyu Y) during the tillering stage was significantly higher than that of the wild type, but the activity of RUBPCase decreased by 30% during the flowering stage, and the activities of SS and SPS were also significantly lower than those of the wild type [35]. At different growth stages of rice, there are significant differences in metabolite expression between the yellow leaf mutant and the wild type.

In this study, ‘ZT00091’ is a stable genetic material of a natural yellow leaf melon mutant. Compared with that of the wild-type ‘ZT091’, the most prominent feature of ‘ZT00091’ is the appearance of yellow leaves beginning at the cotyledon stage, and yellow leaves are evident throughout the growth cycle. The growth cycle is relatively delayed. Therefore, in order to ensure the high and stable yield of melon, exploring the metabolic mechanism of melon yellow leaf mutants is of great significance.

When carotenoid synthesis is hindered, the light energy captured by the photoreaction center decreases, directly weakening the efficiency of light energy conversion. The Z-ISO gene mutation in rice leads to the interruption of carotenoid synthesis, a decrease in photosynthetic pigment content, a reduction in light energy absorption efficiency, and ultimately inhibition of the photosynthetic rate [36]. Carotenoids can prevent photo-oxidative damage by quenching the triplet chlorophyll (^3^ Chl ^+^) in chloroplasts and clearing ROS. Synthetic defects can lead to excessive excitation of the light system and oxidative stress. When the distribution of carotenoids is disrupted, Arabidopsis experiences severe photobleaching under high light intensity [37]. Carotenoids are involved in the assembly and maintenance of the stability of thylakoid membranes, and their absence can lead to disintegration of the photosynthetic membrane system [38]. Wang et al. reported that, compared with normal plants, yellow leaf mutants presented fewer granules and granule thylakoids in their chloroplasts, and the lipid peroxidation of thylakoid membranes was intensified. The stability of photosynthetic protein complexes (such as PSII and PSII) was significantly reduced, which affected the photosynthetic function of chloroplasts [39]. In this study, the cell structures of ‘ZT00091’ and ‘ZT091’ leaves were observed. The mesophyll cells in the chloroplasts of the yellow leaf mutants were infrequent and small, with irregular long spindle shapes and unclear outlines. The grana lamellae were loosely arranged and scattered, with large gaps between lamellae, unclear layers, and even fractures. No starch grains were observed in the chloroplasts. The chloroplast membrane of the melon variety ‘ZT091’ was intact, the structure was neat, and many large starch granules were present. Starch granules are important energy storage substances that provide energy for the growth and morphological stability of leaves. No starch granules were found in ‘ZT00091’, which may explain why the yellow leaf mutant presented narrower leaves and slower growth than did the melon variety ‘ZT091’. This result is generally consistent with previous research results. Therefore, the integrity of chloroplasts may affect the photosynthetic capacity of melon, leading to color changes in melon leaves.

Nontargeted metabolomics is a technique that aims to identify differential metabolic pathways and the differentially abundant metabolites involved [40]. In this study, the differentially abundant metabolites were enriched mainly in the carotenoid biosynthesis pathway, and the excessive accumulation of carotenoids usually induces the production of a yellow leaf phenotype in melon. As a member of the carotenoid family, the metabolism of canthaxanthin is closely related to changes in leaf color. Excessive accumulation of lutein can obscure chlorophyll, resulting in yellow or orange-yellow leaves. The yellowing mutant of melon leaves caused abnormal pigment accumulation due to the reduced activity of carotenoid lyase dioxygenase (CCD), resulting in the sustained yellowing of cotyledons and mature leaves [33]. Monilin, such as *GhSCY2D*, regulates chloroplast development and structural integrity to ensure the normal function of chloroplasts and thylakoid membranes, thereby affecting chlorophyll synthesis and degradation processes. The destruction of the chloroplast structure may lead to increased color development of carotenoids; in contrast, structurally stable chloroplasts can delay chlorophyll loss and maintain leaf greenness [41]. In this study, the carotenoid biosynthesis pathway map revealed that the carotenoid metabolism of canthaxanthin and monilin was significantly downregulated in leaves, which may be the main factor generating changes in melon leaf color.

Wang et al. [42] studied the molecular mechanism of β-carotene accumulation in orange-flesh muskmelons and reported that the relative expression of *CmPDS* and *CmLCY-B* in orange-flesh muskmelons is greater than that in white-flesh muskmelons. The β-carotene lyase gene *CmCCD4* is downregulated in orange-flesh muskmelons and upregulated in white-flesh muskmelons. Excessive expression of *CmCRTR-B* and *CmCRTR-E* promotes lutein biosynthesis in corn. In this study, β-carotene, which is regulated by *CmLCY-B*; α-carotene, which is regulated by *CmLCY-E*; and lutein, which is regulated by *CmCRTR-B* and *CmCRTR-E*, were significantly increased in ‘ZT00091’. In contrast, key genes in the carotenoid degradation pathway, such as *CmCCD4* and *CmNCED3*, were expressed at lower levels in ‘ZT00091’ than in ‘ZT091’ at all growth stages. Therefore, the genes upregulated in ‘ZT00091’ lead to the biosynthesis of carotenoids compared with those in ‘ZT091’, and the downregulated expression of genes weakens the enzymatic activity of chlorophyll in ‘ZT00091’, leading to relative pigment accumulation and yellow leaf mutation. A multiomics study revealed that the downregulation of *CmZEP* expression may lead to plant yellowing [4,43]. In our study, the relative expression of *CmZEP* was significantly downregulated in ‘ZT00091’, which is consistent with previous research results. *CmVDE* and *CmNXS* expression did not significantly differ between ZT00091 and ZT091. This may result in the excessive accumulation of carotene and lutein, leading to yellow leaves in ‘ZT00091’, while *CmVDE* and *CmNXS* mainly regulate the accumulation of violanthin and neoxanthin. Therefore, no significant upregulation of their expression was found in the ‘ZT00091’ yellow leaf mutant, which is consistent with the results of the KEGG metabolic pathway enrichment analysis.

Leaf coloring results from interactions among different pigments are as follows. The chlorophyll and carotenoid contents of the yellow-leaf mutant ‘ZT00091’ were lower than those of the wild-type ‘ZT091’. The chlorophyll content in yellow mutant plants was significantly lower than that in wild-type plants in a study of *Brassica napus*, which is consistent with these results. These findings preliminarily suggest that the formation of the leaf phenotype in yellow leaf mutant plants may be related to a significant decrease in the total chlorophyll content and Chla/b value and a significant increase in the proportion of carotenoids. The results of this study indicated that the carotenoid pathway was significantly enriched and that the mutant leaves turned yellow. Therefore, on the basis of the qRT-PCR results, metabolic pathway changes, and plant phenotype changes, yellow leaves are hypothesized to be related to the carotenoid pathway. The next step will be to analyze metabolite levels further and detect changes in corresponding enzyme activity. This study preliminarily explored the cell structure, metabolomics, and physiological characteristics of melon yellow leaf mutants, laying a foundation for further studies on the mechanisms underlying yellow leaf mutants.

## Figures and Tables

**Table 1:** qRT-PCR primers for differentially expressed genes in the carotenoid pathway.

No.	Accession Number	Gene Name	Forward Primer (5’→3’)	Reverse Primer (5’→3’)
1	NM_001316071	CmLCYE2	TGAGGGAGGATTACGCTGAC	AAAGCACCACCATCACCAAC
2	XM_029091515	CmCCD4	GCAAATGGCATTGGTCTTGC	TATCTCCGTTGGACGTCAGG
3	NM_001247297	CmLCY-B	ATCACTCGTAGCTCGTCCTG	GCTGTACCACCGATTCCAAC
4	NM_112304.3	CmNCED3	TACCTGACCAGCAAGTCGTT	TTGTCGTAAACCACCGGAGA
5	NM_001247166	CmPDS	TCATCAACGTTCCGTGCTTC	TCCATGCAGCTACCTTTCCA
6	NM_001247883	CmPSY1	ACAGGCAGGTCTATCCGATG	GCTCAATTCTGTCACGCCTT
7	NM_001247742	CmPSY2	TTGTGGCGAAGTATGTGCAG	TAGGGCCATCAACAAGCTCA
8	NM_001337545	CmZDS	AGTGCGTTCTAACACCAGGA	TACAACCGACGACCAAGTGA
9	NR_142395.1	CmGGPS1	CCACGACGATCTCCCTTGTA	CACTTTGTGGTTGGTCGGTT
10	NM_127943	CmGGPS2	TCGTCGGAGGAATTGGGAAA	GAAGATGTTGCCGTGCATCT
11	NM_001247373	CmCRTR-E	TCCCAGTGTTCTCGTCCAAA	TCCTCCATCAGGTGTCCCTA
12	PQ_008160	CmCRTR-B	AAAGGCTGATGCCGTTGATG	GCATGTTTGGAGACAGGCAA
13	NM_001309304	CmZEP	TCAGTCGCATGACTTTGCAG	GCACCAACCAGAAGATCACC
14	NM_001318672	CmNXS	CCTAATTCAGGTCGGGCTCA	TGGAAAGTGGTGAAGGGTCA
15	NM_001247681	CmVDE	CCTGCATGTGCAGCTAATGT	ACGAGAACACTGGGATCAGG
16	NM_166556.3	Cmcyc-B	AACTAAACTGGAGCCCGTCA	CCACTCTCTCTCTCGGTGTC

## Data Availability

The data presented in this study are available upon request from the corresponding author.

## Supplementary Materials

The following supporting information can be downloaded at: https://www.mdpi.com/article/10.3390/plants14152300/s1, Table S1, Differentially abundant metabolites, Table S2, Statistical analysis.

## References

[1] Kaleem M.M., Nawaz M.A., Ding X. (2022). Comparative analysis of pumpkin rootstocks mediated impact on melon sensory fruit quality through integration of non-targeted metabolomics and sensory evaluation. Plant Physiol. Biochem..

[2] Chen T., Fu W., Yu J., Feng B., Li G., Fu G., Tao L. (2022). The photosynthesis characteristics of colored rice leaves and its relation with antioxidant capacity and anthocyanin content. Sci. Agric. Sin..

[3] Zhu Y., Yuan G., Wang Y. (2022). Mapping and functional verification of leaf yellowing genes in watermelon during whole growth period. Front. Plant Sci..

[4] Lin N., Gao Y., Zhou Q. (2022). Genetic mapping and physiological analysis of chlorophyll-deficient mutant in *Brassica napus* L.. BMC Plant Biol..

[5] Lin X., Chen X., Wang P. (2022). Metabolite profiling in albino tea mutant *Camellia sinensis* ‘Fuyun 6’ using LC–ESI–MS/MS. Trees.

[6] Chen P., Hu H., Zhang Y., Wang Z., Dong G., Cui Y., Qian Q., Ren D., Guo L. (2018). Genetic analysis and fine-mapping of a new rice mutant, white and lesion mimic leaf1. Plant Growth Regul..

[7] Wu H., Shi N., An X. (2018). Candidate genes for yellow leaf color in common wheat (*Triticum aestivum* L.) and major related metabolic pathways according to transcriptome profiling. Int. J. Mol. Sci..

[8] Liu M., Wang Y., Nie Z. (2020). Double mutation of two homologous genes YL1 and YL2 results in a leaf yellowing phenotype in soybean [*Glycine max* (L.) Merr]. Plant Mol. Biol..

[9] Guan H., Xu X., He C. (2016). Fine mapping and candidate gene analysis of the leaf-color gene ygl-1 in Maize. PLoS ONE.

[10] Zhang T., Dong X., Yuan X., Hong Y., Zhang L., Zhang X., Chen S. (2022). Identification and characterization of CsSRP43, a major gene controlling leaf yellowing in cucumber. Hortic. Res..

[11] Gao J., Ren R., Wei Y. (2020). Comparative metabolomic analysis reveals distinct flavonoid biosynthesis regulation for leaf color development of *Cymbidium sinense* ‘Red Sun’. Int. J. Mol. Sci..

[12] Tang L., Ma X., Zhang Y. (2022). Analysis of differential metabolites in different varieties of Ningxia goji berries based on non-targeted metabolomics. J. Food Saf. Qual. Test..

[13] Shao Q. (2013). Study on a New Melon Leaf Color Yellowing Leaf Mutant. Ph.D. Thesis.

[14] Zheng J., Zhang A., Hu Z. (2019). Genetic Analysis of Leaf Color Yellowing Traits in Melon. Summary of Papers at the 2019 Academic Annual Conference and the 90th Anniversary Commemorative Conference of the Chinese Horticultural Society.

[15] Wang P., Zheng Y., Guo Y. (2020). Widely targeted metabolomic and transcriptomic analyses of a novel albino tea mutant of “Rougui”. Forests.

[16] Wu Y., Guo J., Wang T., Cao F. (2020). Metabolomic and transcriptomic analyses of mutant yellow leaves provide insights into pigment synthesis and metabolism in *Ginkgo biloba*. BMC Genom..

[17] Xinru L., Kuangkuang L., Yuanpeng H., Meiling H. (2025). Distribution, germplasm characteristics, and flower color analysis of wild Shandan in Taigu District. J. Plant Genet. Resour..

[18] Ruoqun H., Jingjing Z., Wanfeng L., Jiayu C. (2025). Joint analysis of transcriptome and metabolome to explore the mechanism of carotenoid synthesis and metabolism in lotus under different shading conditions. Biotechnol. Bull..

[19] Ziwei H., Leichen Z., Yingzi C., Yong Z. (2025). Correlation analysis between carotenoid content and flesh color in watermelon fruit. China Cucurbits Veg..

[20] Luan Y., Fu X., Lu P. (2020). Molecular mechanisms determining the differential accumulation of carotenoids in plant species and varieties. Crit. Rev. Plant Sci..

[21] Wang S., Luan Y., Xu C. (2023). Research progress on esterification of plant lutein. Acta Hortic. Sin..

[22] Isaacson T., Ohad I., Beyer P. (2004). Analysis in vitro of the enzyme CRTISO establishes a polycis-carotenoid biosynthesis pathway in plants. Plant Physiol..

[23] Chen Y., Li F., Wurtzel E. (2010). Isolation and characterization of the Z-ISO gene encoding a missing component of carotenoid biosynthesis in plants. Plant Physiol..

[24] Wang Q., Zhang H., Wei L. (2023). Yellow-green leaf 19 encoding a specific and conservative protein for photosynthetic organisms affects tetrapyrrole biosynthesis, photosynthesis, and reactive oxygen species metabolism in rice. Int. J. Mol. Sci..

[25] Xiong X., Wang Y., Tian H. (2023). Molecular mechanism of yellowing of pumpkin cotyledons based on transcriptome sequencing. Acta Agric. Zhejiangensis.

[26] Wan H., Zhao Z., Qian C. (2009). Selection of appropriate reference genes for gene expression studies by quantitative real-time polymerase chain reaction in cucumber. Anal. Biochem..

[27] Schmittgen T., Livak K. (2008). Analyzing real-time PCR data by the comparative C(T) method. Nat. Protoc..

[28] Lichtenthaler H.K. (1987). Chlorophylls and Carotenoids, Pigments of Photosynthetic Biomembranes. Methods Enzymol..

[29] Wang J., Fan L., Sheng Y. (2019). Analysis of differences in physiological and biochemical indicators of stigma in different flowering stages of melon varieties. Hubei Agric. Sci..

[30] Sun Y., Bai P.P., Gu K.J. (2022). Dynamic transcriptome and network-based analysis of yellow leaf mutant *Ginkgo biloba*. BMC Plant Biol..

[31] Koski V.M., Smith J.H. (1951). Chlorophyll formation in a mutant, white seedling-3. Arch. Biochem. Biophys..

[32] Wang P., Jia R., Yu Q. (2022). Mining of leaf color yellowing related genes in tobacco yellow leaf mutants based on transcriptome sequencing. Mol. Plant Breed..

[33] Feng X., Ma Y., Liang Q. (2025). Deletion of GhSCY2D causes impaired chloroplast development and temperature-dependent leaf yellowing in cotton (*Gossypium hirsutum* L.). Plant Cell Environ..

[34] Yang Y., Jia Y., Feng L. (2023). A point mutation in the gene encoding Mg-chelatase subunit I influences strawberry leaf color and metabolism. Plant Physiol..

[35] Cui H., Yuan B., Wang W. (2014). Carbon metabolism characteristics of rice yellow leaf mutant Huang Yuyue. J. Nucl. Agric..

[36] Ding C., Shao Z., Yan Y., Zhang G., Zeng D., Zhu L., Hu J., Gao Z., Dong G., Qian Q. (2024). Carotenoid isomerase regulates rice tillering and grain productivity by its biosynthesis pathway. J. Integr. Plant Biol..

[37] Hertle A.P., García-Cerdán J.G., Armbruster U. (2020). A Sec14 domain protein is required for photoautotrophic growth and chloroplast vesicle formation in Arabidopsis thaliana. Proc. Natl. Acad. Sci. USA.

[38] Li B., Fu Y., Xi H. (2023). Untargeted metabolomics using UHPLC-HRMS reveals metabolic changes of fresh-cut potato during browning process. Molecules.

[39] Tadmor Y., Burger J., Yaakov I. (2010). Genetics of flavonoid, carotenoid, and chlorophyll pigments in melon fruit rinds. J. Agric. Food Chem..

[40] Xing M., Xue H. (2012). A proteomics study of auxin effects in Arabidopsis thaliana. Acta Biochim. Biophys. Sin..

[41] Zhao J., Yu X., Wang X. (2014). Molecular mechanism of β-carotene accumulation in orange-flesh muskmelons. Shandong Agric. Sci..

[42] Wang Y., He W., Li D. (2019). The effects of different concentrations of NaCl treatment on carotenoid enrichment in germinated corn. Mod. Food Sci. Technol..

[43] Zhou B., Lian Z., Bo Z. (2023). A multi-omics approach identifies bHLH71-like as a positive regulator of yellowing leaf pepper mutants exposed to high-intensity light. Hortic. Res..

